# Correlations of Anthropometric Measurements With Body Fat Percentage, Fat Mass, and Fat Mass Index in School-Age Children

**DOI:** 10.7759/cureus.73597

**Published:** 2024-11-13

**Authors:** Surya Pratap Singh, Mohd Saeed Siddiqui, Pradnya M Joshi, Kiran N Kudlikar, Madhurasree Nelanuthala, Anju M Varghese, Balam Rishitha

**Affiliations:** 1 Department of Pediatrics, Mahatma Gandhi Mission (MGM) Medical College and Hospital, MGM Institute of Health Sciences, Aurangabad, IND

**Keywords:** anthropometric measurements, body fat percentage, body fat prediction, childhood obesity, fat mass, fat mass index, prediction equation

## Abstract

Background: Childhood obesity is a growing public health issue globally, including in India. Anthropometric measures such as body mass index (BMI), waist circumference, and skinfold thickness are commonly used to estimate body fat percentage (BF%), but their correlations with fat mass (FM) and fat mass index (FMI) are less emphasized. This study aimed to explore the relationships between anthropometric measurements and body fat indicators (BF%, FM, and FMI) in school-age children and obtain prediction equations for FM and FMI.

Methods: This observational cross-sectional study included 250 children (125 boys, 125 girls) aged six to 15 years. Anthropometric measures (BMI, waist circumference, skinfold thickness, etc.) and body composition (via bioelectrical impedance analysis) were collected. Pearson's correlation and multiple regression analyses were used to assess the relationships between anthropometric measurements and body fat indicators and to develop prediction models.

Results: BMI, waist circumference, and triceps skinfold thickness showed strong correlations with FM (r = 0.74, r = 0.73, r = 0.61, respectively) and FMI (Pearson's correlation coefficient (r) = 0.76, r = 0.64, r = 0.57, respectively), while the waist-to-height ratio (r = 0.08) and the arm-to-height ratio (r = 0.12) were poorly correlated with BF%. Our prediction equations for FM and FMI provided better predictive values (R² = 0.75 and 0.69, respectively) than BF% (coefficient of determination (R^2^) = 0.35).

Conclusion: FM and FMI showed stronger correlations with anthropometric measurements than BF%. The waist-to-height ratio and the arm-to-height ratio had small correlations with all three body fat indicators. The prediction equation for FM and FMI outperformed the one for BF%, underscoring their potential utility in assessing adiposity in school-age children.

## Introduction

Childhood obesity has become a major public health concern worldwide, with increasing prevalence in both developed and developing countries. From 1975 to 2016, the global age-standardized prevalence of obesity in children and adolescents increased from 0.7% to 5.6% in girls and from 0.9% to 7.8% in boys [1]. India is facing a similar crisis, with the prevalence of childhood obesity rising, particularly in urban areas, ranging between 3.6% and 11.7% [2]. Children face complications from obesity, including cardiometabolic issues, mechanical problems, and psychosocial consequences (including low self-esteem and social difficulties) [3,4]. Childhood obesity significantly increases the risk of adult obesity, premature mortality, and various physical morbidities such as cardiometabolic diseases, type 2 diabetes, cardiovascular diseases, and certain cancers [5-7].

Body mass index (BMI) is commonly used to assess obesity, but its limitations in reflecting body fat distribution highlight the importance of focusing on body fat indices. Excess body fat, rather than BMI, is a better predictor of long-term health risks, including metabolic syndrome and cardiovascular complications [8]. Measuring body fat accurately is critical for understanding the health risks associated with obesity. Dual-energy X-ray absorptiometry (DEXA) is considered the gold standard for measuring body composition, offering precise assessments of fat mass, lean mass, and bone density [9]. However, due to its high cost and limited availability, bioelectrical impedance analysis (BIA) is often used where a weak electric current is passed through the body, and the voltage is measured to calculate the impedance (resistance and reactance) of the body tissues. It is a reliable alternative to DEXA for children [10,11]. While body fat percentage (BF%) is commonly used to assess obesity, fat mass (FM) and fat mass index (FMI) have been found to be independently and positively associated with the presence of metabolic syndrome [12].

Several anthropometric measures, such as BMI, waist circumference (WC), skinfold thickness (SFT), mid-upper arm circumference (MUAC), the arm-to-height ratio (AtHR), and the waist-to-height ratio (WtHR), are often used to estimate body fat percentage and assess obesity-related health risks in children. However, the correlations between these measures and FM or FMI are still unclear, especially in children. This study aimed to explore the correlations of various anthropometric measures with BF%, FM, and FMI and obtain their prediction equations in school-age children in India.

## Materials and methods

Study design and setting

This observational cross-sectional study aimed to identify correlations between various anthropometric parameters and BF%, FM, and FMI. It also sought to obtain prediction equations for estimating these body fat indices using anthropometric measurements in school-age children. The study was carried out at Mahatma Gandhi Mission Medical College and Hospital, Aurangabad, India, from October 2022 to June 2024.

Study participants

The study population comprised healthy school-going children aged between six and 15 years. A total of 250 children participated, with equal representation of genders (125 males and 125 females), who were attending the OPD for minor illnesses and were able to undergo the procedure of BIA. The participants were selected through a stratified random sampling technique to ensure a balanced representation across different age groups and genders. Children with known endocrinal disorders (such as hypothyroidism or Cushing’s disease), chronic conditions (including hypertension, kidney disease, or liver disease), or congenital anomalies were excluded as these conditions may alter body composition.

Sample size estimation

As the literature shows, studies have found varying Pearson's correlation coefficients (r) for anthropometric measurements. To ensure statistical power across a range of expected correlations for anthropometric measurements with a precision of 0.1, a 95% confidence level, and an anticipated 10% dropout rate, the sample size calculations required a minimum of 87 participants for r = 0.75 and 244 participants for r = 0.5 [13,14]. Since these represent the upper and lower correlation estimates, respectively, we chose the rounded higher sample size (n = 250) to ensure proper results across the range of possible correlations [15]. 

Data collection 

Various anthropometric parameters were measured to gather data to identify correlations with BF%, FM, and FMI. Height (cm) was measured using The Concept India (TCI) Star Health Stadiometer (TCI Star Health Products, Mumbai, India) while body weight was recorded to the nearest 0.1 kg using an Omron digital weighing scale (HN-286) (Omron Healthcare, Inc., Kyoto, Japan). BMI was calculated using the standard formula: BMI = weight (kg) / height (m)^2^. The Indian Academy of Pediatrics (IAP) charts for BMI were used to classify overweight (above 23 adult equivalent) and obesity (above 27 adult equivalent). Skinfold thickness was assessed at two specific sites, the calf and the triceps, using Zhart calipers equipped with digital displays (Zhart India, Jaipur, India).

Waist circumference was measured using a non-stretchable measuring tape, and the WtHR was subsequently calculated by dividing WC by height. Additionally, MUAC was measured at the midpoint between the olecranon process and the acromion process, and the AtHR was determined by dividing MUAC by height. To estimate body composition measures such as BF%, visceral fat%, and skeletal muscle mass%, BIA was performed using the Omron Karada Scan HMB-3 device (Omron Healthcare, Inc., Kyoto, Japan). Before measurement using the BIA machine, participants were instructed to adequately hydrate by consuming sufficient water to ensure accurate results. Additionally, all metal objects, including jewelry and watches, were removed to prevent interference with the machine's readings. Participants were then positioned on the footpads of the BIA machine with their feet placed shoulder-width apart and were instructed to grasp the handles provided for support during the measurement process. Fat mass is calculated as BF% * weight (kg)/100 and FMI as FM (kg)/height (m)^2^.

Statistical analysis

Data from the study was entered into an Excel spreadsheet (Microsoft Corporation, Redmond, Washington, United States) and analyzed using jamovi software version 2.4 (The jamovi project (2023), Sydney, Australia) [16]. Descriptive statistics were employed to summarize the data, with categorical variables expressed as frequencies and percentages and continuous variables described using means and standard deviations (SDs). A student's t-test was used to compare continuous variables, and chi-square was used to compare categorical variables. Pearson’s correlation coefficient was calculated to assess the correlation between age, BMI, WtHR, SFT, and AtHR, with BF%, FM, and FMI. A p-value < 0.05 was considered statistically significant. To obtain the prediction equation, we performed multiple regression using the Enter method and selected all anthropometric parameters with statistically significant correlation coefficients. Then we removed those parameters from the model that had a p-value > 0.01 and aimed to obtain a close adjusted coefficient of determination (R^2^) and standard error of estimate (SEE). 

Ethical considerations

The research protocol was reviewed and approved by the institutional ethics committee of Mahatma Gandhi Mission (MGM) Medical College and Hospital, Aurangabad, India (approval number: MGM/PHARMAC/ECRHS/2022/129, dated: September 15, 2022). Informed consent was obtained from the parents or guardians of all participating children, and assent was obtained wherever applicable.

## Results

This study included 250 school-age children (125 males and 125 females), with mean ages of 10.7 years for females (SD = 2.7) and 10.6 years for males (SD = 2.9). There were no significant differences in weight, height, or BMI between genders (Table 1). However, females had a higher mean BF% (mean = 27.6%, SD = 9.7) than males (mean = 24.3%, SD = 10.2; p = 0.01) and significantly greater subcutaneous fat (mean = 17.8%, SD = 6.4 vs. mean = 16.0%, SD = 7.1; p = 0.038). In contrast, males exhibited a higher visceral fat percentage than females (mean = 6.7%, SD = 6.1 vs. mean = 4.4%, SD = 4.0; p = 0.022). FM and FMI were slightly higher in females, though these differences did not reach statistical significance (Table 1).

**Table 1:** Descriptive statistics of the participants BMI: body mass index; SFT: skin fold thickness; MUAC: mid-upper arm circumference; SMM: skeletal muscle mass

Parameter		Female	Male	Total	p-value
		N = 125	N = 125	N = 250	
Age (yrs)		10.7 (2.7)	10.6 (2.9)	10.7 (2.8)	0.757
Weight (kg)		31.7 (11.3)	33.0 (14.1)	32.3 (12.7)	0.415
Height (cm)		140.3 (14.3)	143.5 (17.0)	141.9 (15.7)	0.109
BMI		15.7 (3.6)	15.7 (4.3)	15.7 (4.0)	0.897
Waist circumference (cm)		62.3 (10.7)	62.5 (10.1)	62.4 (10.4)	0.84
Waist-to-height ratio		0.4 (0.1)	0.4 (0.1)	0.4 (0.1)	0.557
Triceps SFT (mm)		18.1 (4.1)	17.6 (5.9)	17.8 (5.1)	0.474
Calf SFT (mm)		19.8 (4.3)	19.4 (5.5)	19.6 (5.0)	0.551
Combined SFT (mm)		37.8 (8.2)	37.0 (11.0)	37.4 (9.7)	0.497
MUAC (cm)		19.0 (3.2)	19.3 (4.0)	19.1 (3.6)	0.581
Arm-to-height ratio		0.1 (0.0)	0.1 (0.0)	0.1 (0.0)	0.717
Classification	Normal	91 (72.2)	71 (57.3)	162 (64.8)	0.047
N (%)	Underweight	23 (18.3)	36 (29.0)	59 (23.6)	
	Overweight	8 (6.3)	7 (5.6)	15 (6.0)	
	Obese	4 (3.2)	10 (8.1)	14 (5.6)	
Body fat %		27.6 (9.7)	24.3 (10.2)	26.0 (10.1)	0.01
Subcutaneous fat %		17.8 (6.4)	16.0 (7.1)	16.9 (6.8)	0.038
Visceral fat %		4.4 (4.0)	6.7 (6.1)	5.4 (5.2)	0.022
SMM %		33.1 (3.9)	33.2 (3.7)	33.1 (3.8)	0.818
Fat mass (kg)		9.3 (5.4)	8.7 (6.4)	9.0 (5.9)	0.472
Fat mass index		4.4 (2.1)	3.9 (2.3)	4.2 (2.2)	0.068

**Figure 1:**
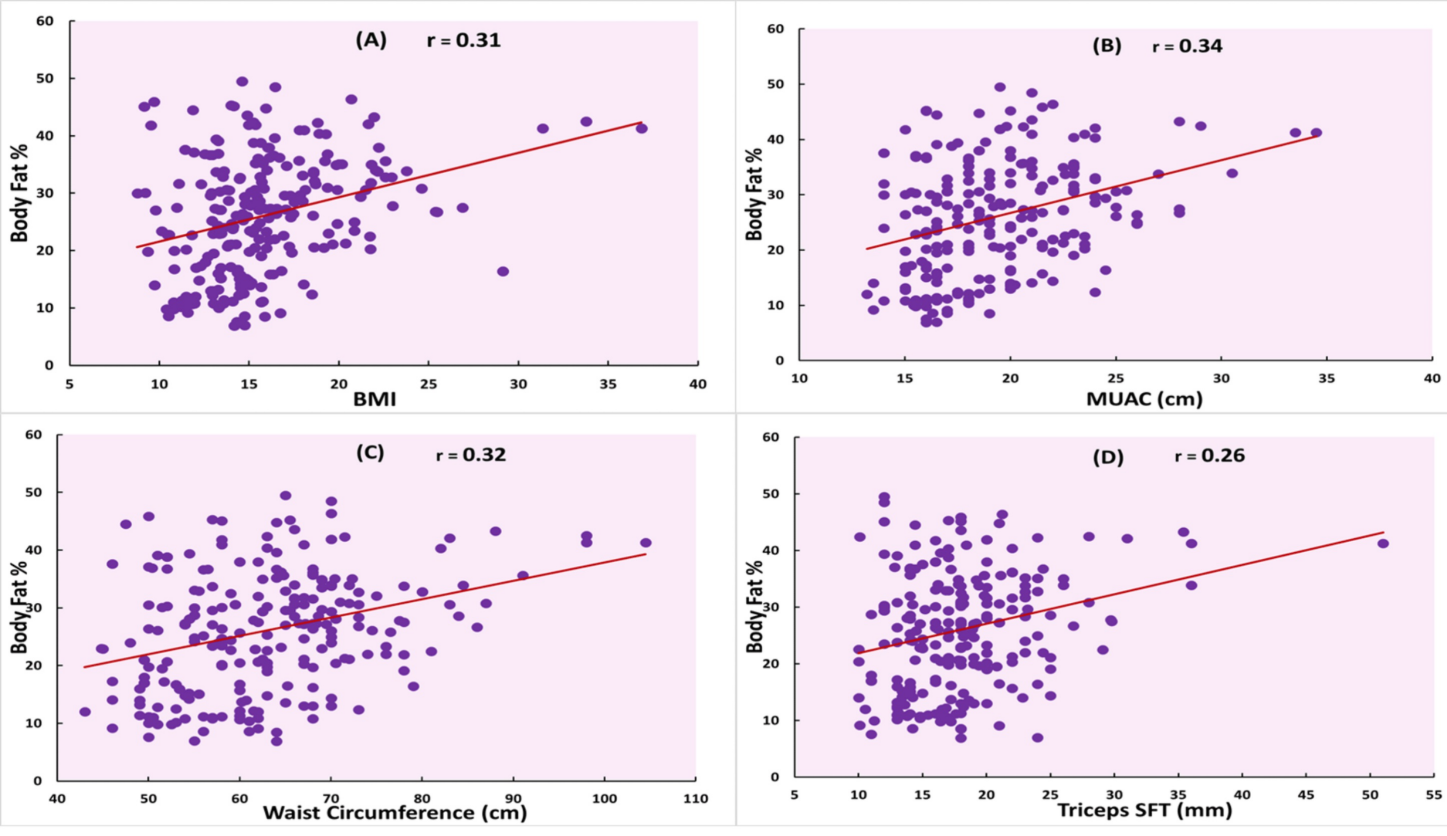
Scatterplots showing correlations of body fat percentage (A) Body mass index (BMI), (B) Mid-upper arm circumference (MUAC), (C) waist circumference, and (D) triceps skin fold thickness (SFT) r: Pearson's correlation coefficient

**Figure 2:**
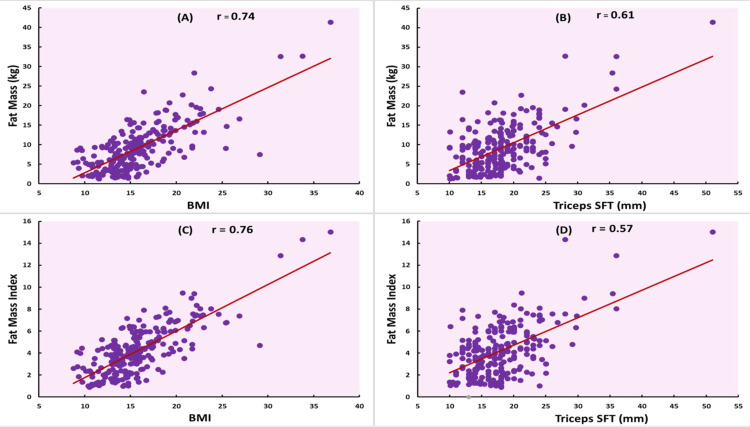
Scatterplots showing correlations of fat mass and fat mass index with body mass index (BMI) and triceps skin fold thickness (SFT) (A) and (B) show the correlations of fat mass with BMI and triceps SFT, and (C) and (D) show the correlations of fat mass index with BMI and triceps SFT.

Age, BMI, WC, and MUAC showed strong correlations with FM and FMI (all p < 0.001). Specifically, BMI had strong correlations with FM (r = 0.74) and FMI (r = 0.76), though its correlation with BF% was moderate (r = 0.31). Similarly, WC was strongly correlated with FM (r = 0.73) and FMI (r = 0.64) but had a weaker correlation with BF% (r = 0.32). MUAC showed strong correlations with FM (r = 0.77) and FMI (r = 0.68) and a moderate correlation with BF% (r = 0.34). In contrast, WtHR and AtHR demonstrated weak correlations with all three body fat indicators, as seen in Table 2. Figures 1, 2 illustrate these relationships, showing higher correlation trends with FM and FMI than with BF%. Figures 1A-1D show scatterplots of correlations of BF% with BMI, MUAC, WC, and triceps SFT, respectively. Figures 2A, 2B show scatterplots of the correlation of BMI and triceps SFT with FM, and Figures 2C, 2D show scatterplots of their correlation with FMI. These findings suggest that these anthropometric measurements were highly correlated with FM and FMI but moderately with BF%.

**Table 2:** Correlation matrix of anthropometric measurements and body fat indices BMI: body mass index; WC: waist circumference; WtHR: waist-to-height ratio; SFT: skinfold thickness; MUAC: mid-upper arm circumference; AtHR; arm-to-height ratio; BF%: body fat percentage; FMI: fat mass index; r: Pearson's correlation coefficient; p: p-value

Variable	Correlation	Age	BMI	WC	WtHR	Triceps SFT	Calf SFT	Combined SFT	MUAC	AtHR	BF%	Fat mass	FMI
Age	r	1	0.46	0.51	0.05	0.3	0.24	0.28	0.59	0.2	0.57	0.71	0.63
	p	..	< .001	< .001	0.422	< .001	< .001	< .001	< .001	0.002	< .001	< .001	< .001
BMI	r	0.46	1	0.73	0.68	0.65	0.63	0.66	0.81	0.78	0.31	0.74	0.76
	p	< .001	..	< .001	< .001	< .001	< .001	< .001	< .001	< .001	< .001	< .001	< .001
WC	r	0.51	0.73	1	0.73	0.72	0.67	0.72	0.83	0.54	0.32	0.73	0.64
	p	< .001	< .001	..	< .001	< .001	< .001	< .001	< .001	< .001	< .001	< .001	< .001
WtHR	r	0.05	0.68	0.73	1	0.55	0.52	0.56	0.51	0.65	0.08	0.39	0.44
	p	0.422	< .001	< .001	..	< .001	< .001	< .001	< .001	< .001	0.215	< .001	< .001
Triceps SFT	r	0.3	0.65	0.72	0.55	1	0.88	0.97	0.71	0.58	0.26	0.61	0.57
	p	< .001	< .001	< .001	< .001	..	< .001	< .001	< .001	< .001	< .001	< .001	< .001
Calf SFT	r	0.24	0.63	0.67	0.52	0.88	1	0.97	0.64	0.53	0.23	0.56	0.53
	p	< .001	< .001	< .001	< .001	< .001	..	< .001	< .001	< .001	< .001	< .001	< .001
Combined SFT	r	0.28	0.66	0.72	0.56	0.97	0.97	1	0.7	0.57	0.25	0.6	0.57
	p	< .001	< .001	< .001	< .001	< .001	< .001	..	< .001	< .001	< .001	< .001	< .001
MUAC	r	0.59	0.81	0.83	0.51	0.71	0.64	0.7	1	0.78	0.34	0.77	0.68
	p	< .001	< .001	< .001	< .001	< .001	< .001	< .001	..	< .001	< .001	< .001	< .001
AtHR	r	0.2	0.78	0.54	0.65	0.58	0.53	0.57	0.78	1	0.12	0.46	0.5
	p	0.002	< .001	< .001	< .001	< .001	< .001	< .001	< .001	..	0.06	< .001	< .001
BF%	r	0.57	0.31	0.32	0.08	0.26	0.23	0.25	0.34	0.12	1	0.76	0.83
	p	< .001	< .001	< .001	0.215	< .001	< .001	< .001	< .001	0.06	..	< .001	< .001
Fat mass	r	0.71	0.74	0.73	0.39	0.61	0.56	0.6	0.77	0.46	0.76	1	0.96
	p	< .001	< .001	< .001	< .001	< .001	< .001	< .001	< .001	< .001	< .001	..	< .001
FMI	r	0.63	0.76	0.64	0.44	0.57	0.53	0.57	0.68	0.5	0.83	0.96	1
	p	< .001	< .001	< .001	< .001	< .001	< .001	< .001	< .001	< .001	< .001	< .001	..

Table 3 shows a summary of the first and second models each for BF%, FM, and FMI. All the significantly correlated predictors, namely, age, gender, BMI, WC, the WtHR, MUAC, the AtHR, triceps SFT, calf SFT, and combined SFT, were included in the first FM and FMI models. However, for BF%, WtHR and AtHR were not included in the first model, as they were not significantly correlated with it. The best predictors for BF% were age, gender, and triceps skinfold thickness (SFT), explaining 35.25% of the variance (R² = 0.35, p < 0.001). For FM and FMI, adding BMI as a predictor improved the model significantly, explaining 74.82% of the variance in FM (R² = 0.75, p < 0.001) and 69.28% in FMI (R² = 0.69, p < 0.001).

**Table 3:** Summary of the multiple regression models R: Pearson's correlation coefficient; R^2^: coefficient of determination; Adjusted R^2^: R^2^ adjusted for the number of predictors; SEE: standard error of estimate; df: degree of freedom

Dependent variable	Model	R	R^2^	Adjusted R^2^	SEE	df	F-statistics	p-value
									Body fat %	Model 1	0.6	0.36	0.34	8.23	8	16.45	< .001
Model 2	0.59	0.35	0.34	8.18	3	43.73	< .001		
Fat mass	Model 1	0.88	0.78	0.77	2.82	10	84.39	< .001
	Model 2	0.86	0.75	0.74	3	4	178.29	< .001
Fat mass index	Model 1	0.84	0.7	0.69	1.25	10	55.56	< .001
	Model 2	0.83	0.69	0.69	1.25	4	135.33	< .001

The final prediction models are as follows:

$\text{BF}\% = 0.12 + 1.96 \times \text{Age (yrs)} + 3.02 \times \text{Gender (female)} + 0.18 \times \text{Triceps SFT (mm)}$

$FM = -15.34 + 0.99 \times \text{Age (yrs)} + 0.29 \times \text{Gender (female)} + 0.57 \times \text{BMI} + 0.26 \times \text{Triceps SFT (mm)}$

$FMI = -4.61 + 0.29 \times \text{Age (yrs)} + 0.45 \times \text{Gender (female)} + 0.29 \times \text{BMI} + 0.06 \times \text{Triceps SFT (mm)}$

These equations apply for both genders where male is the reference (so scored 0) and female is scored 1 in the equation. These models highlight that BMI, WC, and SFT were stronger predictors of FM and FMI than BF%, while the WtHR and the AtHR were weaker predictors.

## Discussion

This study aimed to assess the correlations between anthropometric measurements and body fat indicators in school-age children. Our findings showed stronger correlations between BMI, WC, and SFT with FM and FMI than with BF%. In contrast, the WtHR and the AtHR demonstrated weaker correlations with all body fat indicators. These results suggest that FM and FMI may be better indicators of adiposity than BF%, despite receiving less attention in the literature.

Our findings are consistent with previous studies showing that BMI correlates more strongly with FM and FMI than BF%. Katzmarzyk et al. [13] reported high correlations between BMI and BF% (r = 0.76-0.96) in children aged nine to 11, particularly during puberty. Similarly, studies in Chinese and Japanese children confirm stronger correlations in older children, especially girls [14]. Although BMI is a reliable predictor of FM, its predictive power for BF% remains moderate, as also noted in Vanderwall's study [17].

Waist circumference in our study showed strong correlations with FM (r = 0.73) and FMI (r = 0.64), which aligns with findings by Santos et al., who reported that WC correlates well with DEXA-measured trunk fat (r = 0.85) [18]. However, the WtHR performed poorly in predicting BF% (r = 0.08), differing from studies by Zong et al., which suggested the WHtR is effective in predicting FM and cardiometabolic risk [19].

Our study also found that MUAC had strong correlations with FM (r = 0.77) and FMI (r = 0.68), like previous findings by Lu et al., who demonstrated MUAC’s utility in identifying obesity in resource-limited settings [20]. Meanwhile, the AtHR showed weak correlations with BF% (r = 0.12), suggesting it may be a less effective measure compared to MUAC.

Triceps SFT had moderate to strong correlations with FM (r = 0.61) and FMI (r = 0.57), consistent with the literature. There are reported similar findings, showing a strong correlation between triceps SFT and BF% (r = 0.68) [21]. Gender differences were also apparent, with stronger correlations between SFT and BF% in females (r = 0.81) than in males (r = 0.69) [22]. This may be due to higher BF% in females than in males. Measurement discrepancies, such as between caliper and ultrasound methods [23], highlighted the need for standardized protocols in pediatric SFT assessments.

Our prediction equations for FM and FMI demonstrated stronger predictive accuracy (R² = 0.75 and R² = 0.69, respectively) than for BF% (R² = 0.35). These findings align with Deurenberg et al.'s work [24], which showed BMI, age, and sex predict BF%, but our models outperform when applied to FM and FMI. Similarly, Dezenberg’s study supports the inclusion of skinfold measurements in prediction models, which improved the accuracy of FM estimates (R² = 0.95) [25]. This highlights the utility of FM and FMI as reliable measures in clinical and public health assessments of pediatric adiposity. Our findings further validate the utility of BMI and triceps SFT in predicting FM, aligning with studies showing BMI and WC as reliable predictors when advanced tools like DEXA are unavailable [26]. This supports Cleary et al.'s findings, where the Schaefer equation accurately estimated FM in obese children [27]. Similarly, the meta-analysis by Hudda et al. demonstrated high predictive accuracy (R² = 94.8%) using models with height, weight, age, sex, and ethnicity [28]. Overall, age, BMI, and SFT are good predictors of body fat in pediatric populations, especially when DEXA is not accessible.

Several limitations in our study must be acknowledged. First, our sample was drawn from a limited geographical area, which may reduce the generalizability of the results to other regions. Second, we relied on indirect measures of body fat (BIA), which, while widely used, is not as accurate as direct measures like DEXA. We have not used BMI z-scores, which may have a better correlation with body fat indices in growing children with changes in weight and height. Additionally, we did not assess dietary intake or physical activity levels, both of which are critical determinants of body fat. Also, we have not done external validation of the obtained prediction equation. School health programs may incorporate BMI and SFT measurements into regular health screenings to improve early detection of adiposity in children. Further multi-centric research in the community setting involving a large sample size is needed to develop more precise tools for predicting adiposity across diverse populations. Regional normative data for FM and FMI may be derived through school health programs. Longitudinal studies can also investigate the relationships between lifestyle factors, such as diet and physical activity, changes in body fat over time, and correlations of FM and FMI with comorbidities of obesity.

## Conclusions

Our study revealed that FM and FMI had stronger correlations with anthropometric measurements compared to BF%. We also found that the WtHR and the AtHR had weaker correlations with body fat percentage, FM, and FMI. Our prediction equation for FM and FMI outperformed the one for BF%, underscoring their potential utility in assessing adiposity. By incorporating FM and FMI into routine health assessments, clinicians may enhance the early detection and management of obesity-related risks in school-age children.

## References

[1] Abarca-Gómez L, Abdeen ZA, Hamid ZA (2017). Worldwide trends in body-mass index, underweight, overweight, and obesity from 1975 to 2016: a pooled analysis of 2416 population-based measurement studies in 128·9 million children, adolescents, and adults. Lancet.

[2] Sashindran VK, Dudeja P (2020). Obesity in school children in India. Public Health in Developing Countries - Challenges and Opportunities.

[3] Gurnani M, Birken C, Hamilton J (2015). Childhood obesity: causes, consequences, and management. Pediatr Clin North Am.

[4] Daniels SR (2009). Complications of obesity in children and adolescents. Int J Obes (Lond).

[5] Reilly JJ, Kelly J (2011). Long-term impact of overweight and obesity in childhood and adolescence on morbidity and premature mortality in adulthood: systematic review. Int J Obes (Lond).

[6] Simmonds M, Llewellyn A, Owen CG, Woolacott N (2016). Predicting adult obesity from childhood obesity: a systematic review and meta-analysis. Obes Rev.

[7] Park MH, Falconer C, Viner RM, Kinra S (2012). The impact of childhood obesity on morbidity and mortality in adulthood: a systematic review. Obes Rev.

[8] Bastien M, Poirier P, Lemieux I, Després JP (2014). Overview of epidemiology and contribution of obesity to cardiovascular disease. Prog Cardiovasc Dis.

[9] Kohrt WM (1998). Preliminary evidence that DEXA provides an accurate assessment of body composition. J Appl Physiol (1985).

[10] Kabiri LS, Hernandez DC, Mitchell K (2015). Reliability, validity, and diagnostic value of a pediatric bioelectrical impedance analysis scale. Child Obes.

[11] Kyle UG, Bosaeus I, De Lorenzo AD (2004). Bioelectrical impedance analysis-part II: utilization in clinical practice. Clin Nutr.

[12] Liu P, Ma F, Lou H, Liu Y (2013). The utility of fat mass index vs. body mass index and percentage of body fat in the screening of metabolic syndrome. BMC Public Health.

[13] Katzmarzyk PT, Barreira TV, Broyles ST (2015). Association between body mass index and body fat in 9-11-year-old children from countries spanning a range of human development. Int J Obes Suppl.

[14] Ochiai H, Shirasawa T, Nishimura R (2010). Relationship of body mass index to percent body fat and waist circumference among schoolchildren in Japan--the influence of gender and obesity: a population-based cross-sectional study. BMC Public Health.

[15] Moinester M, Gottfried R (2014). Sample size estimation for correlations with pre-specified confidence interval. Quant Methods Psychol.

[16] (2024). jamovi - open statistical software for the desktop and cloud. https://www.jamovi.org/..

[17] Vanderwall C, Randall Clark R, Eickhoff J, Carrel AL (2017). BMI is a poor predictor of adiposity in young overweight and obese children. BMC Pediatr.

[18] Santos S, Severo M, Lopes C, Oliveira A (2018). Anthropometric indices based on waist circumference as measures of adiposity in children. Obesity (Silver Spring).

[19] Zong X, Kelishadi R, Kim HS (2024). Utility of waist-to-height ratio, waist circumference and body mass index in predicting clustered cardiometabolic risk factors and subclinical vascular phenotypes in children and adolescents: a pooled analysis of individual data from 14 countries. Diabetes Metab Syndr.

[20] Lu Q, Wang R, Lou DH, Ma CM, Liu XL, Yin FZ (2014). Mid-upper-arm circumference and arm-to-height ratio in evaluation of overweight and obesity in Han children. Pediatr Neonatol.

[21] Planinsec J, Fosnaric S (2009). Body mass index and triceps skinfold thickness in prepubertal children in Slovenia. Coll Antropol.

[22] Ahmad M, Ahmed H, Airede KI (2013). Triceps skin fold thickness as a measure of body fat in Nigerian adolescents. Niger J Paediatr.

[23] Lewandowski Z, Dychała E, Pisula-Lewandowska A, Danel DP (2022). Comparison of skinfold thickness measured by caliper and ultrasound scanner in normative weight women. Int J Environ Res Public Health.

[24] Deurenberg P, Weststrate JA, Seidell JC (1991). Body mass index as a measure of body fatness: age- and sex-specific prediction formulas. Br J Nutr.

[25] Dezenberg CV, Nagy TR, Gower BA, Johnson R, Goran MI (1999). Predicting body composition from anthropometry in pre-adolescent children. Int J Obes Relat Metab Disord.

[26] Jensen NS, Camargo TF, Bergamaschi DP (2016). Comparison of methods to measure body fat in 7-to-10-year-old children: a systematic review. Public Health.

[27] Cleary J, Daniells S, Okely AD, Batterham M, Nicholls J (2008). Predictive validity of four bioelectrical impedance equations in determining percent fat mass in overweight and obese children. J Am Diet Assoc.

[28] Hudda MT, Fewtrell MS, Haroun D (2019). Development and validation of a prediction model for fat mass in children and adolescents: meta-analysis using individual participant data. BMJ.

